# Nesting Phenology of Marine Turtles: Insights from a Regional Comparative Analysis on Green Turtle (*Chelonia mydas*)

**DOI:** 10.1371/journal.pone.0046920

**Published:** 2012-10-09

**Authors:** Mayeul Dalleau, Stéphane Ciccione, Jeanne A. Mortimer, Julie Garnier, Simon Benhamou, Jérôme Bourjea

**Affiliations:** 1 UMR Espace-Dev, University of La Réunion, Saint-Denis, La Réunion, France; 2 Kélonia, l’observatoire des tortues marines de La Réunion, Saint-Leu, La Réunion, France; 3 Seychelles Islands Foundation, Victoria, Mahé, Seychelles; 4 Department of Biology, University of Florida, Gainesville, Florida, United States of America; 5 The Zoological Society of London, London, United Kingdom; 6 Centre d’Ecologie Fonctionnelle et Evolutive, Centre National de la Recherche Scientifique, Montpellier, France; 7 Institut Français de Recherche pour l’Exploitation de la Mer de La Réunion, Délégation de l’Océan Indien, Le Port, La Réunion, France; University of Wales Swansea, United Kingdom

## Abstract

Changes in phenology, the timing of seasonal activities, are among the most frequently observed responses to environmental disturbances and in marine species are known to occur in response to climate changes that directly affects ocean temperature, biogeochemical composition and sea level. We examined nesting seasonality data from long-term studies at 8 green turtle (*Chelonia mydas)* rookeries that include 21 specific nesting sites in the South-West Indian Ocean (SWIO). We demonstrated that temperature drives patterns of nesting seasonality at the regional scale. We found a significant correlation between mean annual Sea Surface Temperature (SST) and dates of peak nesting with rookeries exposed to higher SST having a delayed nesting peak. This supports the hypothesis that temperature is the main factor determining peak nesting dates. We also demonstrated a spatial synchrony in nesting activity amongst multiple rookeries in the northern part of the SWIO (Aldabra, Glorieuses, Mohéli, Mayotte) but not with the eastern and southern rookeries (Europa, Tromelin), differences which could be attributed to females with sharply different adult foraging conditions. However, we did not detect a temporal trend in the nesting peak date over the study period or an inter-annual relation between nesting peak date and SST. The findings of our study provide a better understanding of the processes that drive marine species phenology. The findings will also help to predict their ability to cope with climate change and other environmental perturbations. Despite demonstrating this spatial shift in nesting phenology, no trend in the alteration of nesting dates over more than 20 years was found.

## Introduction

Change in phenology, defined here as the timing of seasonal activities of animals and plants [1], is one of the most frequently observed ecological responses to environmental disturbances [1]–[3]. Studies of phenological variations in space and time are keys for understanding and predicting consequences of such disturbances as climate change on living organisms [2], [4]. Marine species are of particular concern since oceans will absorb approximately 80% of the extra warmth [5], impacting fundamental parameters such as sea temperature [6], biogeochemical composition [7] and sea level [8]. Phenological responses to environmental disturbances have already been described in marine species, such as planktonic communities [9], fishes [10] and more specifically in ectothermic animals such as marine turtles [11]–[15].

Reproductive phenology is crucial to the life cycle of marine turtles. First, marine turtles travel considerable distances in short periods between nesting and foraging grounds several times in their lifetime [16]. As adults they tend to show fidelity to both nesting [17] and foraging grounds [18], [19] which may be separated by thousands of kilometres [20]. For example, green turtles *Chelonia mydas* that nest at Ascension Island in the middle of the Atlantic Ocean migrate more than 2300 km to reach their foraging grounds along the Brazilian coast [21]. Second, the seasonal variation in environmental parameters of the beaches where turtles lay their eggs may impact both nesting behaviour and egg-laying success of nesting females [16], [22]. Third, nesting phenology may influence egg clutch survival and hatchling sex-ratio as egg chamber environmental conditions are known to impact egg survival, hatchling emergence and sex determination [23]. Finally, given that newly emerged hatchlings drift mainly passively with ocean currents, their spatial fate and therefore their survival may rely on the time of emergence [24]–[26].

Knowledge of marine turtle phenology is still incomplete [27]. Marine turtle populations display a variety of seasonal nesting patterns. Although most rookeries exhibit a distinct nesting season (example in Godley et al. [28]), with a period of the year without nesting events, other rookeries have year round nesting activity [29]–[35]. Most marine turtle nesting populations exhibit a marked peak, with a greater number of nesting females [27]. This peak usually occurs at the same period of the year but important inter-annual variations in timing and nester abundance are often observed [29], [30]. Environmental stochasticity at foraging grounds has been advanced as a hypothesis to explain these variations [36], [37] with sometimes spatial synchrony between geographically separated rookeries [30]. In the case of the green turtle, an herbivore feeding primarily on seagrasses and algaes, nesting populations show particularly important phenological variations [30], [31] that could be attributed to their relatively low position on the food-chain [36]. Thirdly, in rookeries in the lower latitudes, the nesting peak is typically in summer. Godley et al. [28],Godley et al. [38] suggested that turtles have adapted their nesting seasonality to avoid months where temperature drops significantly.

In the South-West Indian Ocean (SWIO) important green turtle rookeries have been monitored for decades (South-east Africa [39]; Seychelles, Glorieuses, Comores, Madagascar [40]; Seychelles [34], [35], [41], [42]; Europa and Tromelin [43], [44]). Most of these islands host year-round nesting activity, but show one marked nesting peak [31]–[35]. It is notable that in some cases more than one seasonal peak is observed but this generally concerns small rookeries like d’Arros Island, Seychelles [34] or Juan de Nova [45]. Remarkably, the SWIO rookeries do not share a single nesting peak. The peak occurs in austral summer on some rookeries [31] and early in the austral winter on others [32]–[35].

Based on long term data sets for green turtle nesting activity, we conducted a regional study focused on the SWIO region to investigate the processes that drive spatio-temporal variation of marine turtle nesting phenology. We present here an analysis of the distribution of nesting peaks across the region over the last two decades relative to environmental conditions. First, the spatial arrangement of nesting peaks was analysed followed by the inter-annual variations in nesting phenology within and between the main green turtle nesting sites. Finally, we investigated the impact of environmental variables, particularly sea surface temperature, on nesting phenology.

## Materials and Methods

### Ethics

This study did not involve any direct manipulation of animals. No specific permits were required for access to beaches for this study. All work done on beaches with restricted access was performed by individuals trained and authorized by the relevant authorities. Study protocol followed the IUCN MTSG standards [46].

### Study Sites and Datasets

**Figure 1:**
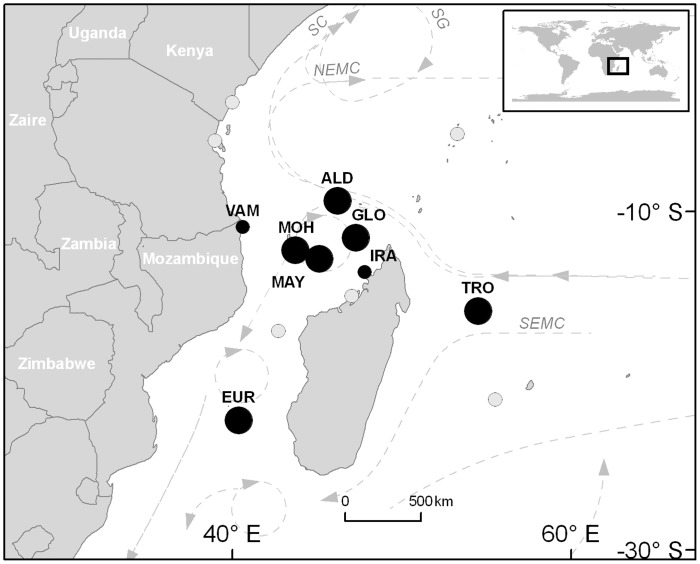
Green turtle *Chelonia mydas* major nesting rookeries in the South West Indian Ocean. Larger circles represent most important rookeries (see associated references in **Table 1**). Black circles are those actually included in the study. Dotted lines represent the main ocean currents in the region [72].

Our study focused on six of the most important green turtle rookeries of the SWIO region (35°S–5°S; 20°E–60°E) located on remote oceanic islands: Europa (EUR), Aldabra (ALD), Tromelin (TRO), Mayotte (MAY), Mohéli (MOH) and Glorieuses (GLO), as well as on two minor rookeries, located on continental coasts: Iranja (IRA) in Madagascar, and Vamizi (VAM) in Mozambique (**Figure 1**). Over the eight rookeries, a total of 21 different sites were monitored: two sites for EUR, GLO, TRO and ALD, five for MOH, six for MAY, one for IRA and one for VAM. These rookeries are spread along large gradients of latitude (22°S to 9°S) and longitude (40°E to 54°E). TRO, off the northeast coast of Madagascar, and EUR, South of the Mozambique Channel, are respectively the most eastern (54°E) and southern (22°S) rookeries. MOH, MAY, GLO and ALD are located at the northern end of the Mozambique Channel with ALD being the most northerly (9°S). Each site has adequate protective legislation although the intensity of effort varies among countries. EUR, GLO, TRO, ALD are remote islands sparsely populated by temporary workers, completely protected, with low level of disturbance and no significant human impact on nesting turtles, while MAY, MOH, IRA and VAM are inhabited islands with intense monitoring programmes and a significant level of turtle protection.

**Table 1:** Origin and characteristic of the nesting green turtle activity datasets used in this study.

Rookery name	Rookery label	Country	References	Counting method	Site names	Period	Targeted Monitoring effort
Europa	EUR	France TAAF	[31], [43], [44]	Crawls	EUR1	1984–2010	Daily
					EUR2	2001–2010	Daily
Glorieuses	GLO	France TAAF	[31]	Crawls	GLO1	1987–2010	Daily
					GLO2	1999–2010	Daily
Tromelin	TRO	France TAAF	[31], [43], [44]	Crawls	TRO1-2	1986–2010	Daily
Mayotte	MAY	France	[33]	Crawls	MAJ1-4, MAO, SAZ	1998–2010	Daily
Aldabra	ALD	Seychelles	[35], [42]	Crawls	SETT	1995–2010	At 2–3 day intervals
					WGT22	1995–2010	At 10 day intervals
Mohéli	MOH	Comoros	[47]	Crawls	BWE, ITS, MIA1-2, TSA	1999–2010	Daily
Iranja	IRA	Madagascar	[32]	Nests	IRA	2000–2010	Daily
Vamizi	VAM	Mozambique	[49]	Nests	VAM	2003–2007	Daily

Long term datasets of nesting activity were compiled in this study. Depending on the abundance of nesting females, nesting activity was recorded in two different ways: beach crawls or nests counting. For the 6 major rookeries, monitoring comprised early morning counts of crawls (i.e., turtle tracks on the beach made during nesting emergences during the night). Three of them (EUR, TRO, GLO) were monitored daily for more than 20 years respectively since 1984, 1986 and 1987 (see detailed protocol in Lauret-Stepler et al. [31]). Two others (MAY and MOH) were monitored daily respectively since 1998 and 1999 (see detailed protocols in Bourjea et al. [33] and Bourjea et al. [47]). The last one (ALD) has been monitored intermittently between 1981 and 1994, and consistently since 1995 (see detailed protocols in Mortimer et al. [35]). On average, monitoring at ALD occurred at 2–3 days intervals at site SETT, and at 10 days intervals at site WGT22. For all sites, we made the assumption that track counts were proportional to female abundance over time [48]. For the 2 minor rookeries (IRA and VAM) nests monitoring was conducted respectively during 2003–2010 and 2003–2007 periods. Due to the low level of nesting activity, nest monitoring consisted of a daily count of the number of nests (see detailed protocols in Bourjea et al. [32] and Garnier [49]). Counting method, frequency, time range, effort and respective references are summarized in **Table 1**.

### Data Interpolation

Daily series of crawl numbers were interpolated as follows. For each time period with missing data, we computed three indices: the number of missing days, the mean number of crawls before and the mean number of crawls after the time period with missing data. The size of sample period for the means calculation being equal to half the number of missing days (*e.g.* if 14 days were missing, means were computed from the seven days preceding and the seven days following the missing gap). However, for this approach, sample periods were limited to a minimum of 5 days and a maximum of 15 days (*e.g.* if 40 days are missing, sample periods were 15 days before and after). For each day with missing data, we then calculated an expected value using a simple linear regression between the mean number of crawls before and the mean number of crawls after the gap. Missing data were finally filled in by random sampling from a Poisson distribution with parameter equal to that expected value. This approach allowed us to estimate missing data taking into account the magnitude of the series as well as the trend in the neighbourhood of the gap. Poisson sampling was intended to avoid artificial monotony in the time series. Missing gaps exceeding 90 days were not interpolated. Such large gaps, due to site accessibility difficulties, occurred on rare occasions. **Table 2** summarizes the percentage of missing data for each nesting site. Each day without counting was considered as one missing data.

**Table 2:** Overall mean peak date and mean peak dispersion (in days) for the 22 monitored nesting sites from 8 green turtle rookeries of the South West Indian Ocean.

Rookery label	Site label	Missing data (%)	Interpolated data (%)	Peak date (Angular mean)	Peak date (Linear mean)	Peak dispersion (Angular deviation)
**EUR**	EUR1	10	90.3	15-Dec	15-Dec	58
	EUR2	20.1	100	21-Dec	18-Dec	61
**TRO**	TRO1	17.3	40.8	20-Jan	21-Jan	60
	TRO2	17.3	40.6	24-Jan	26-Jan	61
**GLO**	GLO1	9.5	87.7	06-May	07-May	69
	GLO2	14.2	82.1	03-May	06-May	63
**MAY**	MAJ1	27.4	96.0	14-Jun	21-Jun	71
	MAJ2	17.4	96.8	26-Jun	01-Jul	70
	MAJ3	17.5	96.8	16-Jun	16-Jun	70
	MAJ4	17.6	96.9	21-Jun	23-Jun	70
	MAO	17.8	96.9	03-Jul	04-Jul	70
	SAZ	11.6	95.3	11-Jun	12-Jun	71
**MOH**	BWE	23.9	23.1	03-Jun	05-Jun	74
	ITS	24.0	23.3	16-Jun	16-Jun	70
	MIA1	24.0	23.4	06-Jun	09-Jun	74
	MIA2	23.9	22.9	19-May	24-May	75
	TSA	23.8	22.8	05-Jun	07-Jun	74
**ALD**	SETT	52.3	91.7	19-Apr	20-Apr	66
	WGT22	88.9	–	06-Jun	08-Jun	75
**IRA**	IRA	–	–	09-May	11-May	73
**VAM**	VAM	–	–	09-Apr	16-Apr	71

In the case of WGT22, the number of missing data was considered too great, so no interpolation was done. For VAM and IRA, due to the low level of nesting activity and since only nesting events were recorded, days with missing data were considered as days without nesting events.

The performance of our interpolation method was estimated by comparing with other interpolation methods (see supporting Information).

After computing all datasets, we ran three sets of analyses, presented below, at different time series frequencies using R statistical software [50].

### Overall Intra-site Nesting Profiles

Firstly, we analysed the nesting phenology over the available monitoring periods by using daily time series compiled for each site into an overall annual intra-site nesting profile. Since all eight rookeries exhibit year round nesting, we could not use standard methods applicable to populations that exhibit distinct nesting seasons. We therefore calculated, for each day of the year, the mean number of crawls over the monitoring period. Each daily time series was then compiled into an overall annual nesting profile. For each of them, we then used circular statistics [51] to compute the angular mean and deviation of nesting peak date associated to each nesting site. In order to use standard statistics methods to perform inter-sites and environmental factors analyses, we compared angular mean with linear standard mean (see formula in [28]).

### Inter-annual Variability of Crawls Time Series

Secondly, we analysed the annual correlation in nesting phenology between the nesting sites by computing annual time series. As nesting occurred all year round there was no evident distinction between the nesting seasons. We then divided the daily time series into annual subsamples by taking six months before and six months after the peak mean nesting date. Each annual subsample was then considered as a single nesting season for which we calculated (1) the median nesting date and (2) the mean number of crawls. These parameters were then considered as annual time series and were handled using time series analyses.

Spatial synchrony in the abundance of nesting turtles between nesting sites was evaluated using cross-correlation function analysis with log transformed variables. As time series exhibited a significant auto-correlation at lag 1, variables were pre-whitened using residuals derived from a first order auto-regression AR(1) model fitted to time series. Finally, to identify which sites shared similar nesting phenology, we conducted hierarchical clustering analyses using cross-correlation results as similarity indices between beaches.

### Relation of Environment with Nesting Profiles

Finally, we studied the relation between nesting phenology and environmental variables using annual time series. Two types of environmental factors were studied: meteorological factors and oceanographic factors. Meteorological datasets consist of air temperature *in-situ* measurements recorded at local weather stations. For the French islands (EUR, TRO, GLO, MAY), these data were available through the French national weather agency (Météo France). For Aldabra, air temperature data were provided by the Seychelles Islands Foundation and the Seychelles Meteorological Bureau [52]. For the other rookeries, no meteorological data were available.

For oceanographic data, we used two sea surface temperature (SST) datasets. For regional SST profile we used a remote sensing dataset obtained from NASA’s satellite AQUA equipped with the Moderate Resolution Imaging Spectroradiometer (AQUA-MODIS). This dataset had a 4 km resolution and was downloaded from the Physical Oceanography Distributed Active Archive Center of NASA at http://oceancolor.gsfc.nasa.gov/. It was used for the regional analysis of the annual nesting profiles. It reflects the regional sea surface annual thermal profile in the SWIO. Annual SST time series, from 1983 to 2010, were computed by aggregating values from daily SST AVHRR-only dataset [53]. This dataset has a spatial grid resolution of 0.25° and was downloaded at http://coastwatch.pfel.noaa.gov/coastwatch/CWBrowserWW180.jsp. We used the AVHRR SST for inter-annual time series analysis since AQUA-MODIS data had insufficient time coverage. In both datasets, to compute annual SST for each island we used a 100 kmx100 km or 1°×1° square buffer around the islands.

A simple linear regression was used to relate nesting activity at each rookery to SST in the vicinity of the rookery. To run the regression, as SST data do not reach the resolution of the nesting site, we computed a single mean nesting date for each rookery by averaging mean nesting date over the sites. We then applied the Moran’I test to check for spatial auto-correlation in the regression residuals [54]. Finally, to investigate inter-annual relationship between the annual nesting activity time series and the environmental time series (Air temperature, SST), we used time series cross-correlation analyses on annual time series. As for synchrony analysis, since crawls time series exhibited a significant auto-correlation, variables were pre-whitened using residuals derived from a first order auto-regression AR(1) model fitted to the time series.

## Results

**Figure 2:**
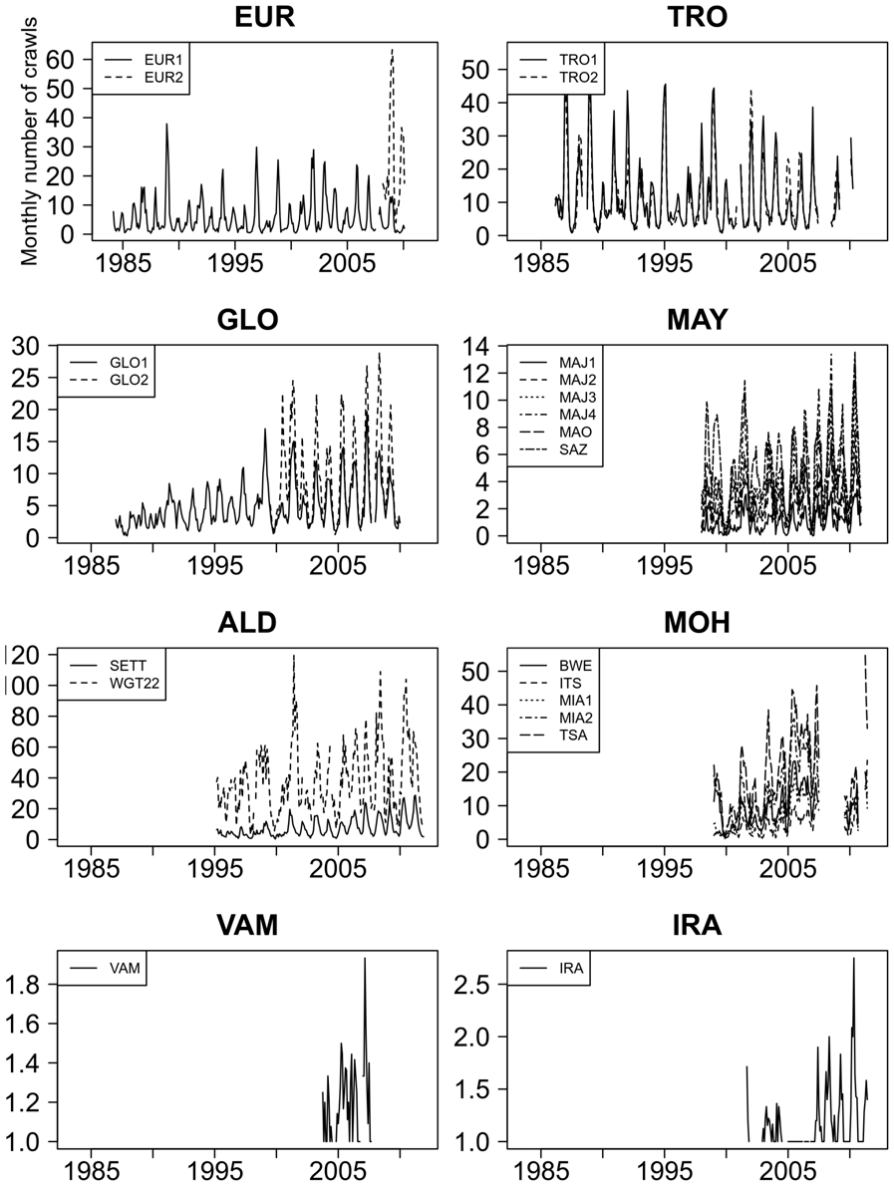
Monthly time series after interpolation of missing values on daily time series. Except for VAM and IRA, the y-axis is the number of counted crawls that were printed on the beach by potential nesters. For VAM and IRA, the y-axis is the number of nests.

The nesting activity dataset used in this study represents more than 80,000 beach patrols at 21 sites from eight rookeries over a time period that ranges from 27 years in EUR to five years in VAM (average 15.5 years, SD = 7.0; **Table 1**). Missing data represented 24.1% of all data (SD = 18.2) and the interpolation allowed the handling of an average of 68.2% (SD = 33.5) of the daily missing values (**Table 2**). The final time series used for this study are represented in **Figure 2**. Best interpolation results were obtained for sites of MAY and ALD (SETT) as the missing gaps rarely exceeded 90 days. For the French Eparses islands (EUR, TRO, GLO), only an average of 73.6% of the missing data could be filled in, mostly because missing gaps often exceed 90 days due to the isolation of these rookeries.

### Nesting Profiles, Median Nesting Date and Dispersion

**Figure 3:**
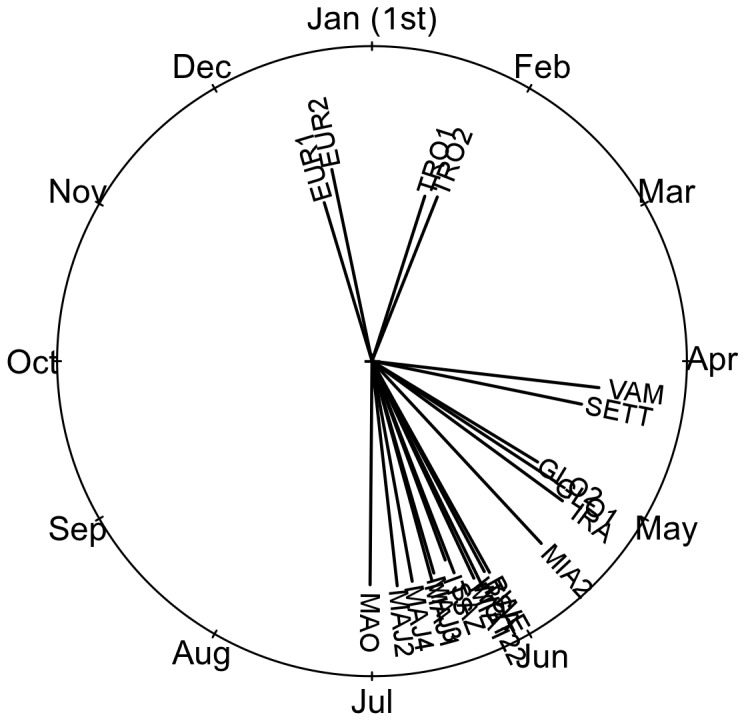
Circular representation of angular mean peak date for the 21 sites. Segment length is proportional to angular deviation around the mean peak date.

Overall intra-site angular mean nesting date and deviation over the monitored periods for each nesting site are summarized in **Table 2** and **Figure 3**. As already highlighted in the literature there is a great variability in the median nesting date between the sites of the SWIO region [31]–[33], [35]. EUR and TRO respectively located in the South and outside the Mozambique Channel, exhibit a peak during the austral summer (December and January) whereas the other rookeries show peak at the beginning of the austral winter between April and June.

Mean nesting dates showed low intra-rookery variability (**Table 2**). For example, EUR, TRO and GLO had a difference of respectively 6, 4 and 3 days between the earlier and the later mean nesting date. Aldabra is the only rookery where the peak nesting dates of two sites geographically separated by only 3 km showed a difference of 48 days between sites.

Results showed that mean nesting peak date over all sites was restricted between December and July (**Figure 3**). By using October 1^st^ as a reference to avoid any artificial cut between December and July, no major differences were found between angular mean and standard linear mean (**Table 2**), we therefore run standard linear statistics methods. We found a strong correlation between the mean peak date and the mean peak dispersion (Spearman ρ≈0.44; p-value = 0.046). Sites that showed a median peak date in austral summer have a less dispersed peak (*e.g.* EUR1∶58 days; **Table 2**) whereas the sites that have a median nesting peak later in the year displayed a larger peak (*e.g.* MAJ1∶71 days; **Table 2**).

Finally, we observed a significant latitudinal gradient, with northern sites exhibiting peaks later in the year (R^2^≈0.71; p-value<0.008), with for example ALD, the northern rookery, showing the earliest peak date of all northern sites.

### Importance of Temperature at Regional Scale

**Figure 4:**
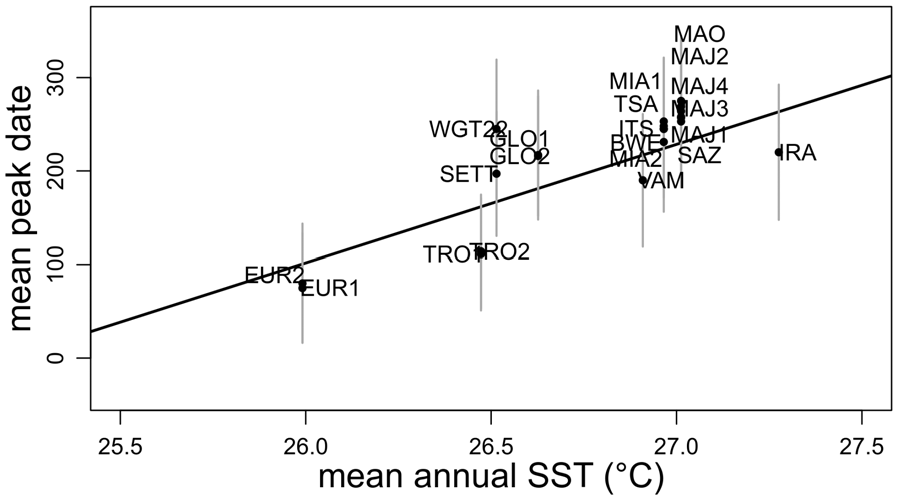
Linear regression of mean peak date against mean annual SSTs.

**Figure 5:**
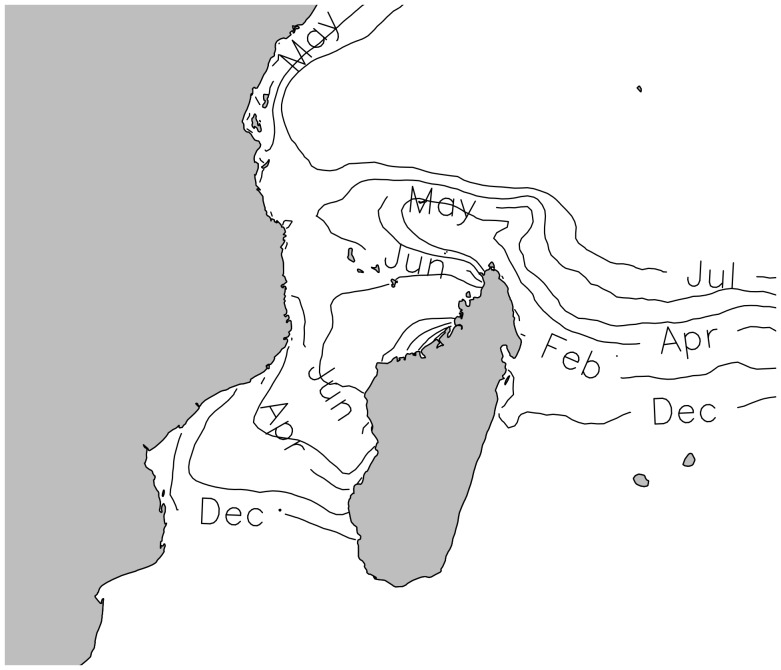
Mapping of mean peak dates smoothed contours according to our SST model.

Overall intra-site mean nesting date is significantly correlated to annual mean SST (R^2^≈0.61; p-value<0.022; **Figure 4**). We found no significant spatial auto-correlation in the regression residuals (Moran’I value = 0.983, p-value = 0.163). Moreover, when excluding the minor rookeries from the analyses (IRA, VAM) as well as WGT22 that was irregularly monitored, the quality of the SST model increases significantly (R^2^≈0.76; p-value<0.005), suggesting a strong relation between the mean annual SST and the mean nesting peak date. Northern and warmer sites tend to show a peak later in the year. Nevertheless sites from rookeries at the extreme north like ALD and GLO have an earlier peak than MOH and MAY. Using the smoothed SST regional map and a linear regression with mean peak date, we mapped the expected mean peak date at regional scale (**Figure 5**).

### Regional Synchrony

EUR2, IRA and VAM were not included in the time series analysis of inter-annual variations due to the short monitoring period and/or the sparse nesting activity recorded (**Table 1**, **Figure 2**). WGT22 was also excluded because of the relatively low sampling effort.

**Figure 6:**
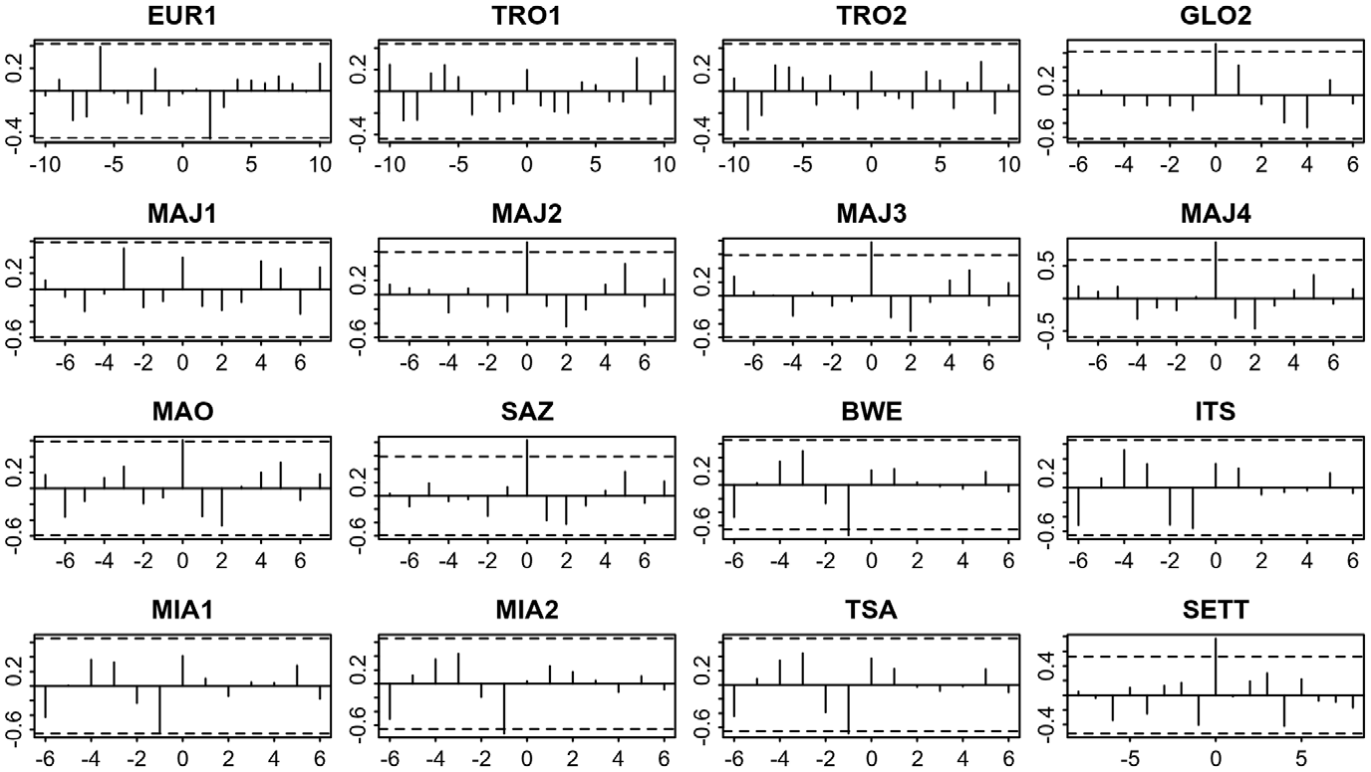
Cross-correlation analysis of median nesting date time series for GLO1 against the other sites. (x-axis: lag; y-axis: correlation coefficient; dotted lines: 95% confidence intervals). The most significant coefficients appear at lag 0 and are positive.

**Figure 7:**
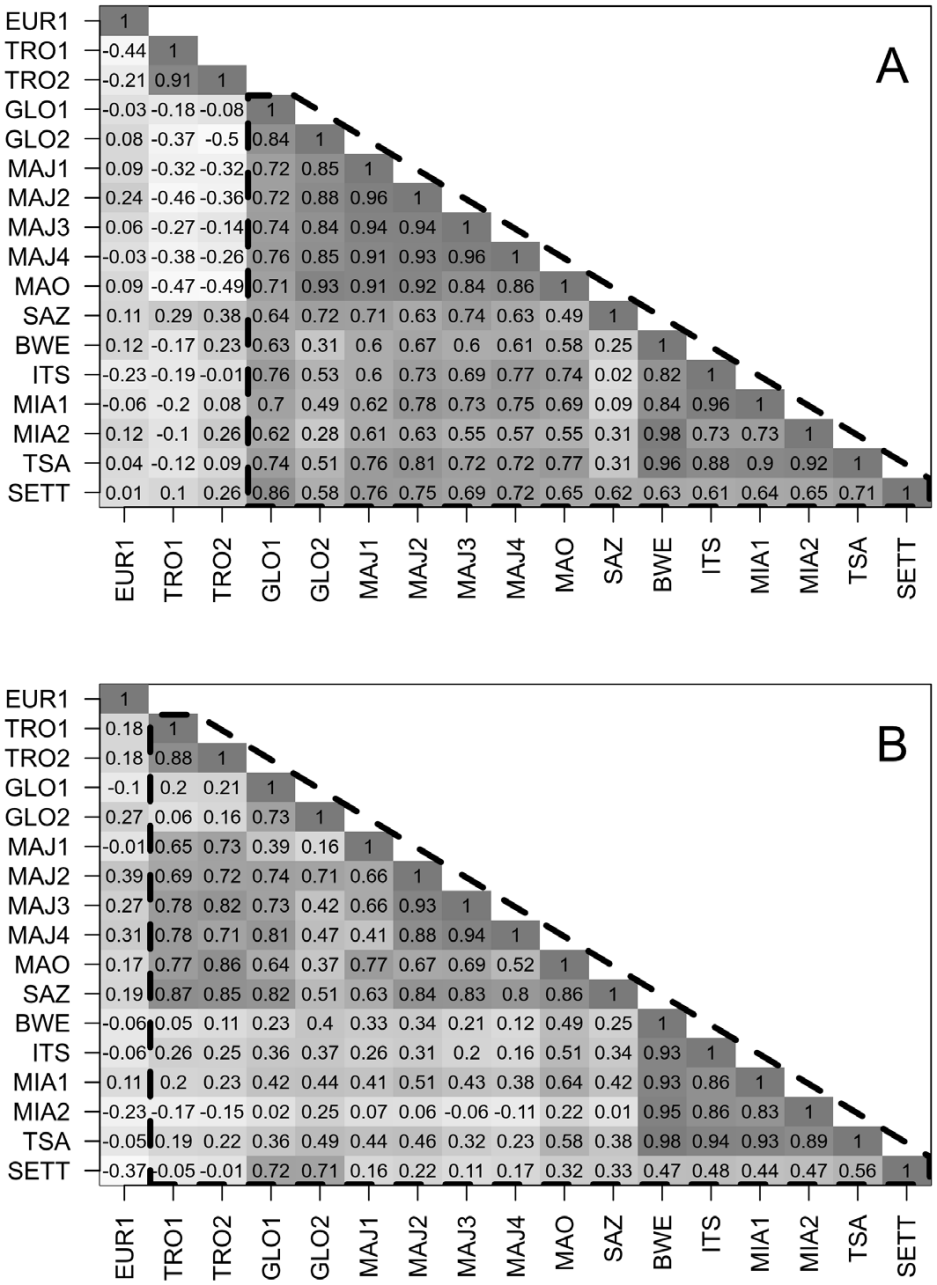
Matrices of cross-correlation at lag 0. Panel A: matrix based on annual mean number of crawls. Panel B: matrix based on median nesting date. Darker cells have higher positive coefficients. Dashed polygons gather sites which were highly correlated between each other.

As regularly observed in marine turtles [30], [36], there is an important inter-annual variability in the nesting profiles regarding both the annual number of nesting females (overall SD = 3.0 crawls.days^−1^) and the median nesting date (overall SD = 26.2 days). Time series cross-correlation analyses between the annual time series for both parameters suggest a spatial synchrony. For most cross-correlations, the most significant coefficient appeared at lag 0, *i.e.*when no lag was introduced between annual crawls time series (**Figure 6**). We then focused only on correlation at this lag. For mean annual number of crawls and median annual date, we found the same trend with overall positive correlations (**Figure 7**).

**Figure 8:**
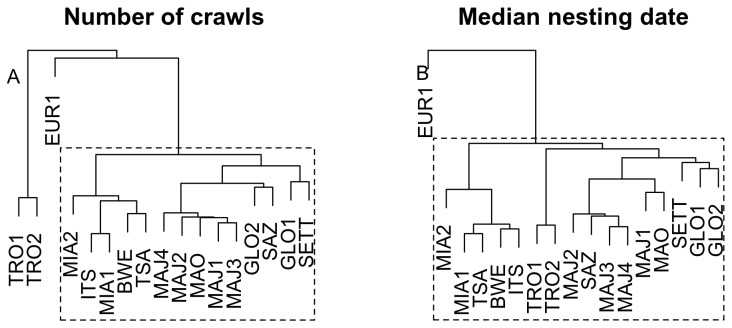
Hierarchical cluster analysis based on cross correlation matrices at lag 0 (see **Figure 7**). Panel A: Hierarchical clustering based on annual mean number of crawls. Panel B: Hierarchical clustering based on annual median nesting date.

Regarding the annual number of crawls, there were strong positive correlations between sites from rookeries located in the northern part of the Mozambique Channel (**Figure 7**A). For example, there are strong correlations between sites from MAY and MOH in the archipelago of Comoros that are separated by less than 100 km. Observed number of crawls on GLO, were also correlated to number of crawls in ALD and MAY, these rookeries being separated by less than 300 km. Beaches from distant islands, EUR1, TRO1 and TRO2 are basically uncorrelated between each other, neither are they significantly correlated with the other nesting beaches. Regarding annual median nesting dates, we also found positive correlation between time series of geographically close islands from the northern part of the Mozambique Channel (**Figure 7**B). Interestingly, correlation was also positive between these islands and the distant rookery TRO, suggesting a common dynamic in term of date of nesting (**Figure 7**B). EUR1 annual median nesting date still appeared uncorrelated with all other beaches. Results from hierarchical cluster analyses using either the correlation matrix based on the mean number of crawls or the correlation matrix based on median nesting dates highlight these findings (**Figure 8**).

### Relation with Temperature

**Figure 9:**
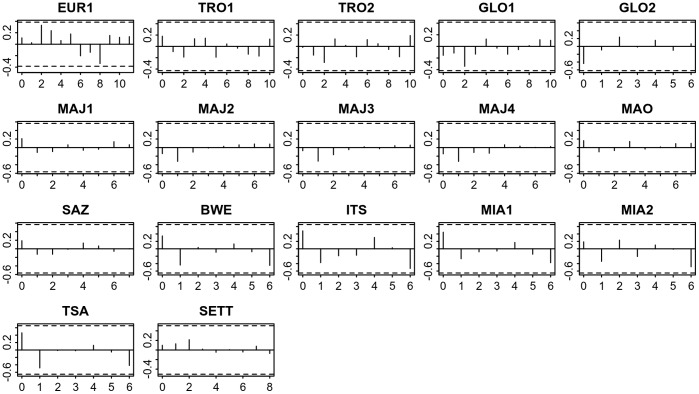
Cross-correlation analysis of annual median nesting date time-series and mean annual SST. (x-axis: lag; y-axis: correlation coefficient; dotted lines: 95% confidence intervals).

Cross-correlation analyses, between crawls annual time series and annual temperature time series (*in-situ* measurement air temperature and telemetry based SST) did not show any particular temporal relation between the annual temperature and nesting activity. Results for median nesting date and SST are presented in **Figure 9**. No cross-correlation coefficients were significant and there was no common pattern in the cross-correlograms. The same remarks also apply to annual mean number of crawls and air or sea surface temperature (results not shown). Therefore, there was no clear evidence that SST or air temperature affects the number of nesting females or the timing of nesting either directly or with any delay.

## Discussion

### Spatial Shift of Nesting Peak Along SST Gradient

The SWIO hosts some of the most important green turtle populations in the world, most of them recovering from human exploitation during past decades [31], [33], [35], [47]. Not all rookeries display similar temporal nesting dynamic and similar seasonality patterns. This variability in nesting peak dates had already been demonstrated for this region [31], [33], [35]. Mortimer et al. [35] noticed a latitudinal gradient with southern sites having a peak in austral summer and northern sites in austral winter. Such ecological changes in phenology along a latitudinal gradient were already observed in a context of global changes studies [55]. However, latitude itself does not explain all observed nesting patterns. For example, the peak at Tromelin located quite high in latitude (15.8°S) appears approximately one month after the peak at Europa located 6.5° further south (22.3°S) and nearly six months before Mayotte located only 3° further north (12.8°S). Moreover northern rookeries like Glorieuses and Aldabra have an earlier peak than Mayotte and Mohéli despite being located at higher latitude. Here we were able to go further in the understanding of nesting peak distribution by comparing SST and nesting peak dates at large spatial scale and we demonstrated that SST distribution shapes regional timing of green turtle nesting in the SWIO. We observed a clear shift of the peak towards the austral winter along SST gradients. The expected peak dates mapped at regional scale (**Figure 5**) contribute to understanding the phenological patterns observed in the SWIO. The two rookeries that exhibit a peak during the austral summer (Europa and Tromelin) share similar and cooler mean annual SST. Glorieuses and Aldabra are exposed to cooler mean annual SST than Mayotte and Mohéli despite being further north thus explaining the earlier nesting peak date.

A similar situation occurs in the western Pacific Ocean [30]. Chaloupka [30] showed a relatively surprising nesting peak during the austral winter in the Australian Gulf of Carpentria while other Australian rookeries exhibit a peak in austral summer. He proposed that these differences in nesting activity could be attributed to differences in genetic structure. This is not the case in the SWIO. First of all, Europa and Tromelin, despite sharing similar nesting seasons, belong to separate genetic stocks [56]. In the same way, Tromelin and Glorieuses belonging to the same stock display opposite nesting seasonality. As the Gulf of Carpentria is exposed to warmer SSTs as compared to other Australian rookeries, it should be interesting to investigate whether SST explains the seasonal nesting patterns.

In rookeries with distinct nesting seasons, those parts of the year without nesting events have been considered as too cold for marine turtle reproduction [28], [38]. Knowledge about the existence and persistence of peaks in year-round rookeries remains scarce [27], [57]. Our spatial analysis suggests that high temperature may also be a factor limiting nesting activity. Despite observing a clear regional distribution of the peak along the SST gradient, we did not detect any temporal trend in the median nesting date nor a correlation with SST. Direct influence of SST on marine turtle timing of nesting is still under debate with contradictory findings. Pike [15] did not find any relation between timing of nesting and SST at nesting for green turtles in Florida, despite the fact that such a relationship has been demonstrated in the same region by Weishampel et al. [12], or on loggerheads turtles (*Caretta caretta*) in other rookeries [12], [58], [59]. If such trend exists in the SWIO, our datasets did not allow to detect it. Direct *in situ* SST measurements (e.g. meteorological stations, oceanographic buoys…) would rather be used but such datasets were not available at our study sites.

### Spatial Synchrony in Nesting Activity

By investigating spatial synchrony in nesting activity in the SWIO, we highlighted that nesting sites of the northern part of the Mozambique Channel (Mohéli, Mayotte, Glorieuses, Aldabra) exhibit a significant inter-annual correlation both in term of timing of nesting and in term of nesting activity. However, Europa exhibited different patterns from all the other nesting rookeries of the region. Spatial synchrony between rookeries has been regularly observed in marine turtles [29], [30]. This suggests that for green turtles an overall spatio-temporal dynamic at regional scale is driven by environmental factors. According to our present knowledge of the green turtle adult cycle, environmental conditions at foraging grounds might strongly influence the observed nesting phenology [16] as in the Atlantic Ocean [60] or in the Mediterranean Sea [36]. The same occurs in other marine turtle species. Chaloupka et al. [37] showed that, for two loggerheads population in the Pacific, there is an inverse correlation between nesting abundance and mean annual sea surface temperature in a foraging region during the year prior to the nesting season. This suggests that turtle nesting in the northern islands of the Mozambique Channel may share common foraging grounds or at least foraging grounds affected by similar environmental dynamics. The positive yet smaller correlation between Tromelin nesting dates and the ones from the north of the Mozambique Channel suggests that turtles nesting in Tromelin may also have overlapping foraging grounds. Another possibility could be that foraging grounds used by green turtles nesting in Europa differ significantly from the ones used by turtles from other rookeries. These hypotheses corroborate with an on-going tracking program in the region.

### The Role of Temperature in Regional Nesting Phenology

Temperature is certainly one of the most crucial environmental factors to marine turtle reproductive biology [37], [60]–[62]. At nesting sites, temperature and other environmental constraints may act on three different life stages (see review in Miller [16]). (1) Temperature might play a role on adult nesting activity and nesting peak might occur during conditions that are more conducive to nesting [63]. (2) Nesting peak might occur when temperature is favourable to embryonic development and survival [28]. (3) Lastly, the nesting peak might occur to allow optimal environmental thermal conditions at hatchling emergence [64]. Most probably, nesting peak conditions are a combination of these three factors.

Temperature at foraging grounds may also affect nesting phenology of marine turtles (see review in Hawkes et al. [65]). Temperature may influence food availability, especially for green turtles which mainly feed on seagrasses [36]. As ectotherms, the physiological state of marine turtles may also be directly dependent on temperature and thus on their ability to start a nesting migration [60] [66]. Lastly temperature could also act as a cue to initiate a nesting migration similar to the photo-period control phenological traits of birds (see review in Dawson et al. [67]). The observation at nesting sites of a correlation and a shift of the nesting peak date along a STT gradient may be a consequence of a similar thermal gradient at foraging grounds. Further investigations in other regions where similar phenological patterns are observed should help clarifying this question.

We observed three patterns in the SWIO: a regional shift of the nesting peak date along a SST thermal gradient at nesting sites, a spatial synchrony of nesting activity (peak date and abundance) between some rookeries, but an absence of inter-annual relation between nesting activity and air or sea surface temperature at nesting sites. These results suggest that thermal conditions at nesting peak influence overall regional nesting phenology with nesting taking place more frequently when thermal conditions are favourable for reproductive success. Inter-annual dynamics may rely more on other environmental factors either at nesting site or at foraging grounds. The identification of the extent of foraging grounds used by each separate population of green turtle in the region should be a key issue to confirm this hypothesis as current knowledge remain scarce to date [68], [69].

### Other Factors Influencing Nesting Phenology

Other factors may also directly or indirectly influence marine turtle phenology. The spatial pattern in SST is closely linked with ocean circulation in the region [70](**Figure 1**). The SWIO is mainly influenced by the westward South Equatorial Current (SEC) [71] and Tromelin is directly exposed to this current. When reaching the North-East of Madagascar, the SEC splits into the Southeast and Northeast Madagascar Currents [72]. Aldabra and Glorieuses are the first rookeries facing this latter flow that brings cooler water. In the other side of Madagascar, the Mozambique Channel is a dynamic area swept by intermittent train of southward large anti-cyclonic eddies [73]. Such an influence of oceanic conditions on SST pattern is visible on the nesting peak mapping drawn using smoothed annual SST (**Figure 6**). We therefore suggest here that nesting phenology observed in the SWIO is consequently driven by this particular hydrodynamic system through its influence on SST.

Our regional analysis suggests that temperature is the main factor that shape nesting phenology of green turtles in the SWIO. Under this hypothesis, when nesting does not occur predominantly during the warmer month of the year (like in all the northern sites), we expect to see a bimodal peak: one during temperature fall and another during temperature rise. Yet, we observed only a single marked peak when temperature goes down in early austral winter. Various hypotheses may explain the absence of a second marked peak. (1) Temperature is not the only factor and other environmental factors might be more dominant. Humidity has been demonstrated to be another key factor for embryonic and hatchling survival [22], [23] and might be a factor influencing nesting site selection [74]. In our study sites, austral winter corresponds to a dry season [31], [33], [49], [52]. We may not observe a second marked peak in late winter and early summer because humidity conditions might not be conducive to successful nesting. (2) As incubation lasts approximately between 50 and 90 days, the overall temperature to which eggs are exposed might be too high, especially at the end of the embryonic development [75]. (3) The physiological condition of individuals following a winter season might be such that females cannot sustain the demands that nesting imposes. (4) The second peak might exist but may be hard to detect because of year round nesting. Bimodal nesting seasons for marine turtles have already been observed in the Atlantic on Leatherback turtle *Dermochelys coriacea* [76], and also on green turtle minor rookeries in the SWIO [34], [45]. Making a statistical distinction between monomodal or plurimodal peaks in nesting profiles can be somewhat subjective.

### Importance of Phenological Response in a Global Warming Context

While warming in the tropical regions might be less pronounced than in temperate areas [5], green turtles as ectotherms might be particularly sensitive to temperature shift [77]. Phenological change may be one of the strategies evolved in response to changes of temperature. Our study demonstrated that green turtle nesting phenology is highly variable in a small geographic area according to a spatial thermal gradient and provides valuable information on the possible response of green turtle to climate change. As an example, the evolution of sex-ratios has been of importance to marine turtles which are a temperature-dependent sex determination species [78], [79]. A possible effect of global warming could then be an adaptation of the mean nesting date resulting in the reduction of any negative impact on sex ratio. Since the northern and warmer sites exhibit a nesting peak at the beginning of the winter season (April-June), in the context of global warming one can then expect a delay in the mean nesting date towards the austral winter. Under such hypothesis, warmer rookeries like islands of the Comoros archipelago (Mohéli and Mayotte) are particularly vulnerable since the peak has already occurred during winter in June and may not be able to shift the nesting period more towards the dry season.

Despite demonstrating a spatial shift in nesting phenology, we did not observe any trend in the mobility of nesting dates over more than 20 years.

Considering marine turtles particular life history, assessing the adaptive capacity and resilience of marine turtles to climate change is challenging [65]. Despite recent proofs of environmental responses, little is known about the biological mechanisms that drive the nesting phenology of marine turtles. And yet these processes are the main factors in determining the rate of adaptation to environmental changes. Characterizing these mechanisms and the life stage at which they occur is required to understand the role of phenotypic plasticity and genetic adaptation in the determination of the nesting phenology and therefore will be a key to assess how rapidly green turtles can adapt to environmental changes.

## Supporting Information

Figure S1 Interpolation method. We tested our interpolation method by comparing its predictive power with three additional methods: (1) sampling randomly in the range of the time series, (2) sampling randomly from the values neighbouring the missing data, (3) interpolating linearly between the values neighbouring the missing data. To assess the predictive power of the method we randomly removed 20% of the values of each time series and we compared the predicted values of each method to the known values. As a comparison measurement, we used the mean absolute deviation indices (MAD), *i.e.* the mean of the absolute difference between the predicted and the exact values. To allow comparison between time series the MAD was scaled by the overall mean value of the time series. We studied the influence of the size of the missing data by increasing the number of consecutive missing values removed from 1 to 90 by step of 5 (19 levels). For the four interpolation methods, the 21 sites and the 19 levels of consecutive missing values, we ran 100 simulations and we computed the mean, the 0.975 and the 0.025 confidence intervals of the scaled MAD. All interpolation methods performed relatively well with the exception of the method sampling randomly in the range of the time series that did not account for neighbouring values (2.840<MAD<3.734). Linear interpolation was the method with the lowest deviation to the exact values (0.384<MAD<0.601; see also Figure S2). Linear interpolation with Poisson sampling performance was intermediate (0.463<MAD<0.684; see also Figure S2). Method of sampling from neighbouring values had the largest deviation among the interpolation methods that takes into account the neighbouring values (0.519<MAD<0.859; see also Figure S2). While linear interpolation was the best method in term of predictive power, this method introduces a strong artificial autocorrelation and do not reflect the stochasticity of the time series. Based on the fact that MAD range between linear with and without and Poisson sampling were both very low, we therefore chose to use linear interpolation with Poisson sampling as it appears to be the best trade-off in term of prediction power and stochasticity.
(TIFF)

Figure S2 Detailed view of Figure S1.
(TIFF)
