# Supplementary figures and images for: Nesting Phenology of Marine Turtles: Insights from a Regional Comparative Analysis on Green Turtle (Chelonia mydas)
Source: PLoS One. 2012 Oct 9;7(10):e46920. doi: 10.1371/journal.pone.0046920 (PMC3467270; doi:10.1371/journal.pone.0046920)

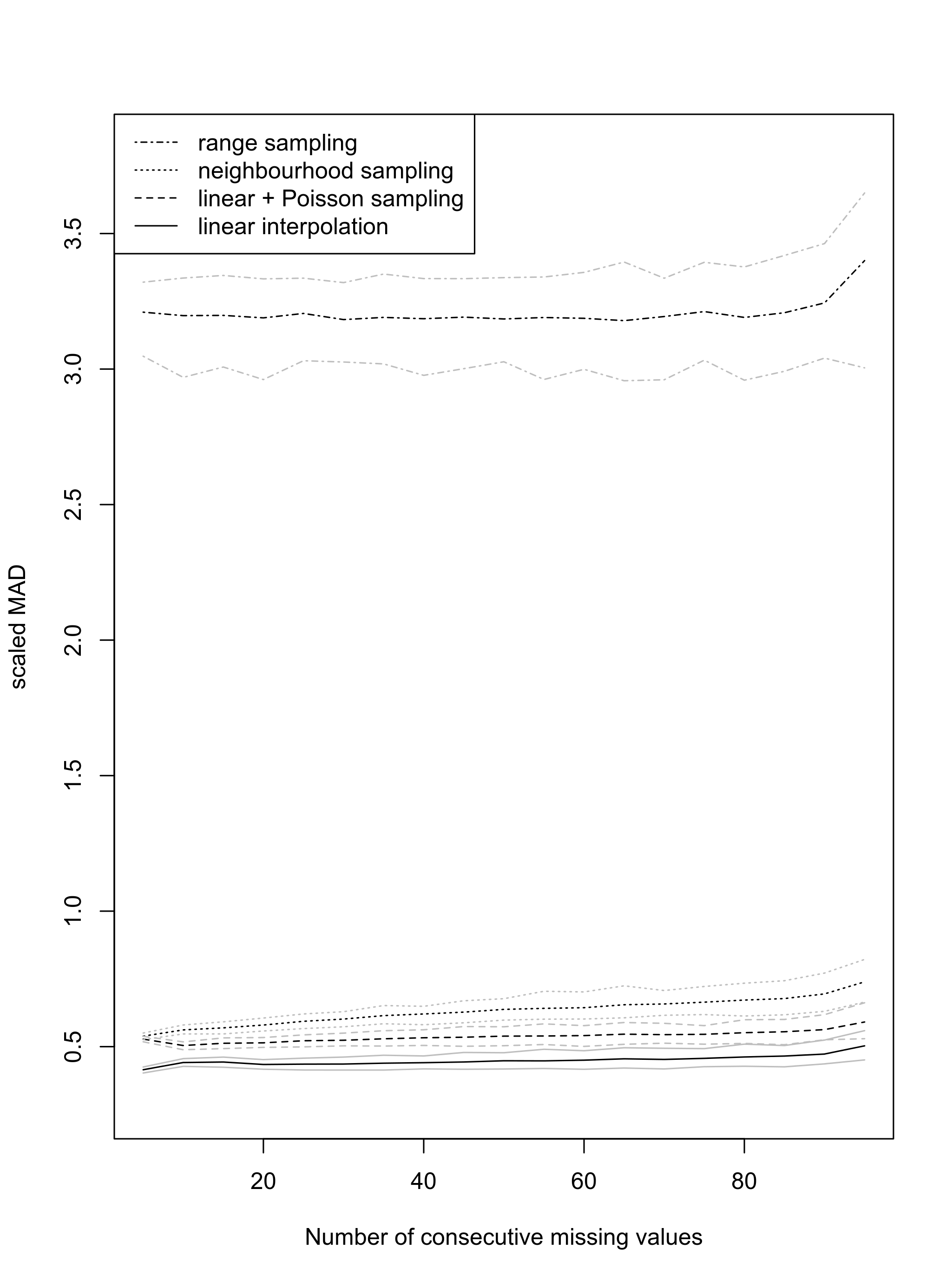

Supplement: Figure S1 — Interpolation method. We tested our interpolation method by comparing its predictive power with three additional methods: (1) sampling randomly in the range of the time series, (2) sampling randomly from the values neighbouring the missing data, (3) interpolating linearly between the values neighbouring the missing data. To assess the predictive power of the method we randomly removed 20% of the values of each time series and we compared the predicted values of each method to the known values. As a comparison measurement, we used the mean absolute deviation indices (MAD), i.e. the mean of the absolute difference between the predicted and the exact values. To allow comparison between time series the MAD was scaled by the overall mean value of the time series. We studied the influence of the size of the missing data by increasing the number of consecutive missing values removed from 1 to 90 by step of 5 (19 levels). For the four interpolation methods, the 21 sites and the 19 levels of consecutive missing values, we ran 100 simulations and we computed the mean, the 0.975 and the 0.025 confidence intervals of the scaled MAD. All interpolation methods performed relatively well with the exception of the method sampling randomly in the range of the time series that did not account for neighbouring values (2.840<MAD<3.734). Linear interpolation was the method with the lowest deviation to the exact values (0.384<MAD<0.601; see also Figure S2). Linear interpolation with Poisson sampling performance was intermediate (0.463<MAD<0.684; see also Figure S2). Method of sampling from neighbouring values had the largest deviation among the interpolation methods that takes into account the neighbouring values (0.519<MAD<0.859; see also Figure S2). While linear interpolation was the best method in term of predictive power, this method introduces a strong artificial autocorrelation and do not reflect the stochasticity of the time series. Based on the fact that MAD range between linear w [file pone.0046920.s001.tiff]

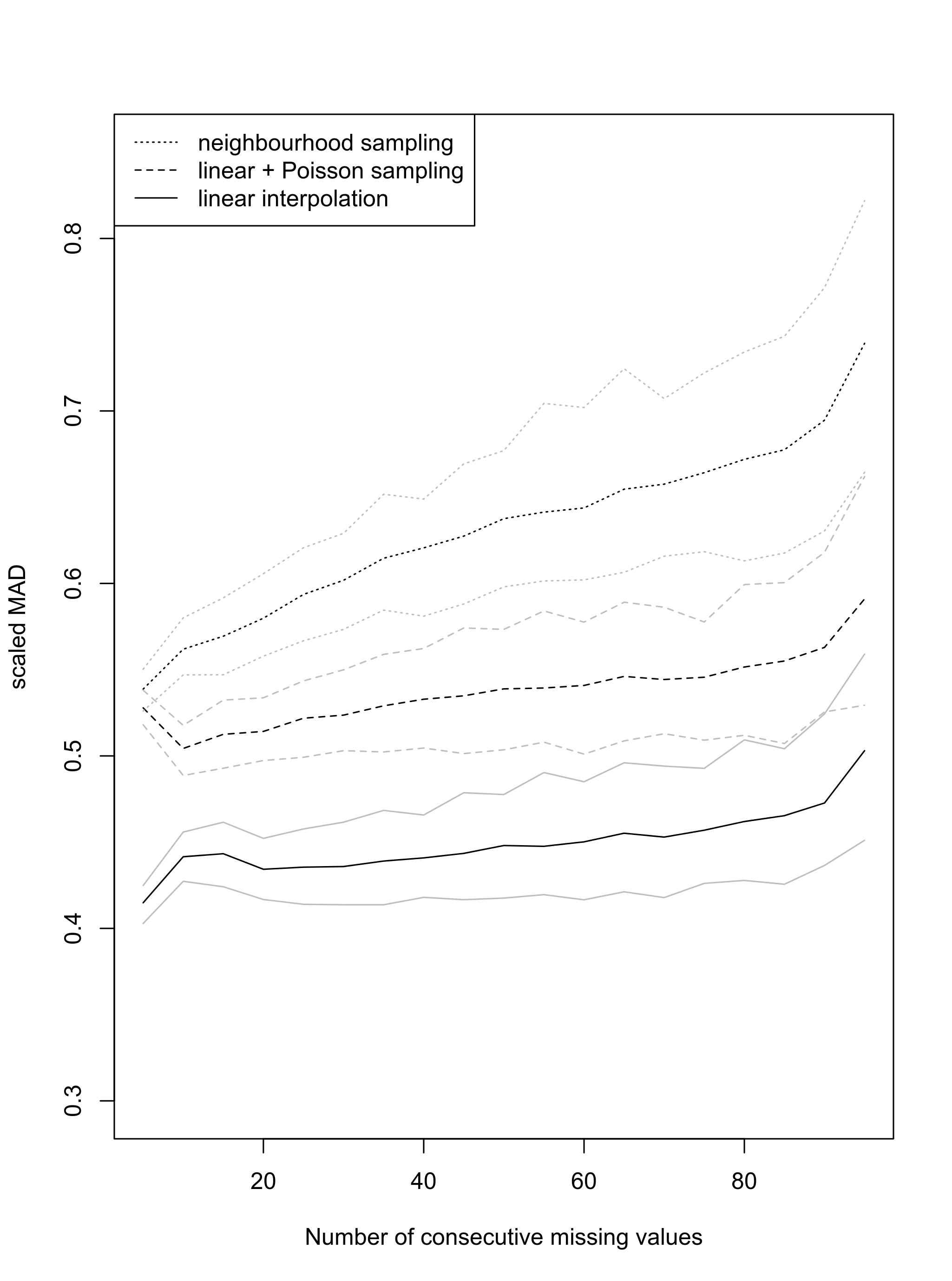

Supplement: Figure S2 — Detailed view of Figure S1. (TIFF) [file pone.0046920.s002.tiff]
